# **Draft genome sequence of** ***Pasteurella multocida*** **strain BD1769 with untypable capsular serotype isolated from a layer chicken**

**DOI:** 10.1128/MRA.00594-23

**Published:** 2023-10-25

**Authors:** Kyohei Hamada, Sachiko Kawashima, Kaori Hoshinoo, Yohsuke Ogawa, Yuichi Ueno

**Affiliations:** 1 Fukuoka Prefectural Chuo Livestock Hygiene Service Center, Fukuoka, Japan; 2 Fukuoka Prefectural Hokubu Livestock Hygiene Service Center, Kama, Fukuoka, Japan; 3 National Institute of Animal Health, National Agriculture and Food Research Organization (NARO), Tsukuba , Ibaraki, Japan; 4 Hokkaido Research station, National Institute of Animal Health, National Agriculture and Food Research Organization (NARO), Sapporo, Hokkaido, Japan; University of Maryland School of Medicine, Baltimore, Maryland, USA

**Keywords:** *Pasteurella multocida*, pasteurellosis, fowl cholera

## Abstract

We report the draft genome sequence of *Pasteurella multocida* strain BD1769 (GenBank accession numbers JARFTQ010000001–JARFTQ010000021) isolated in 2021 from a layer chicken in Japan. No gene locus for capsular biosynthesis was annotated in the genome of this strain.

## ANNOUNCEMENT

*Pasteurella multocida* is a Gram-negative, facultative, anaerobic, extracellular bacterium that causes pasteurellosis in birds (fowl cholera) and mammals, including humans (1). *P. multocida* strains are grouped into five capsular serotypes (A, B, D, E, and F) using an indirect hemagglutination test with type-specific antisera (2, 3). These serotypes have also been determined through PCR targeting the capsular biosynthesis gene locus (4). No capsular serotype has been reported to be more or less virulent than others, but serotype A has been the most frequently isolated serotype in cases of avian pasteurellosis (1, 5).

In September 2021, an increase in mortality and a decrease in the egg-laying rate were observed in a poultry farm in Japan. We isolated bacteria from the lung, liver, spleen, heart, and kidney of three chickens by stamping them on 5% sheep blood agar and incubating at 37°C under 5% CO_2_ for 24 h. Smooth, non-motile, non-hemolytic colonies of Gram-negative coccobacilli were observed on agar stamped with the lungs, livers, and kidneys. Although the colonies lacked the mucoid features usually observed on *P. multocida* colonies, these isolates were confirmed as *P. multocida* using a commercial biochemical identification kit, ID-test HN-rapid 20 (Nissui, Japan), and *P. multocida*-specific PCR (6). Capsular serotype of these isolates could not be identified using an indirect hemagglutination test (7) or a multiplex PCR targeting the capsular biosynthesis gene locus (4). We designated one of the liver isolates as BD1769.

For DNA extraction, BD1769 was cultured using the same method as that used for bacterial isolation. Genomic DNA was extracted from agar streak using Puregene Yeast/Bact. Kit (Qiagen, Germany) according to the manufacturer’s instructions. The genomic DNA was fragmented to 350-bp using Covaris LE220 ultrasonicator (Covaris, USA). DNA library was constructed using TruSeq DNA PCR-free library prep kit (Illumina, USA). Fragment size was checked using Agilent 2100 bioanalyzer (Agilent, USA). DNA concentration was measured using Qubit fluorometer (Thermo Fisher Scientific, USA) and NanoDrop spectrophotometer (Thermo Fisher Scientific). Sequencing was performed by Macrogen Japan Corporation using the Illumina NovaSeq 6000 platform (Illumina) with 150-bp paired-end reads.

Illumina sequencing yielded 33,657,644 reads, with a total sequenced nucleotide (nt) count of 5,082,304,244 nt. The total reads were trimmed using Platanus_trim version 1.0.7 (http://platanus.bio.titech.ac.jp/pltanus_trim) with default parameters and assembled using SPAdes version 3.14.0 (parameters: --careful -k 21,33,55,77,99,127,133,141,145,149) (8). The assembly yielded 24 scaffolds. We removed contigs shorter than 200 nt manually, the length of the remaining 21 scaffolds was 2,265,117 nt in total, *N*_50_ value was 354,087 nt, and GC content was 40.34%. The average coverage was 2,156×. Genome annotation was performed using Prokaryotic Genome Annotation Pipeline version 6.4 (9) (National Center for Biotechnology Information), and the scaffolds were composed of 2,085 protein-coding genes, 6 complete rRNAs, and 51 tRNAs. No gene locus for capsular biosynthesis was annotated in the genome sequence of this strain.

## Data Availability

The draft genome sequence of *P. multocida* BD1769 is available in GenBank under accession numbers JARFTQ010000001 to JARFTQ010000021 . The raw sequence data are available under Sequence Read Archive (SRA) accession number DRX428319 (BioProject accession number PRJDB15269 ).
